# Position-dependent changes in phycobilisome abundance in multicellular cyanobacterial filaments revealed by Raman spectral analysis

**DOI:** 10.17912/micropub.biology.000799

**Published:** 2023-05-01

**Authors:** Jun-ichi Ishihara, Yuto Imai

**Affiliations:** 1 Medical Mycology Research Center, Chiba University; 2 Faculty of International Politics and Economics, Nishogakusha University

## Abstract

The one-dimensional filamentous cyanobacterium, *Anabaena* sp. PCC 7120, shows a simple morphological pattern consisting of two distinct cell types under nitrogen-deprived conditions. We found that microbial pigment composition in differentiated (heterocyst) and undifferentiated cells (vegetative cells) can be distinguished using Raman microscopy. The Raman bands associated with phycocyanin and allophycocyanin were of higher intensity in vegetative cells than those in heterocysts. However, these bands had statistically lower intensity in vegetative cells located further away from heterocysts. That is, the pigment composition in individual cells is affected by locational information in a filament.

**Figure 1. Raman band intensities of phycocyanin and allophycocyanin in individual cells are affected by locational information in a filament.**
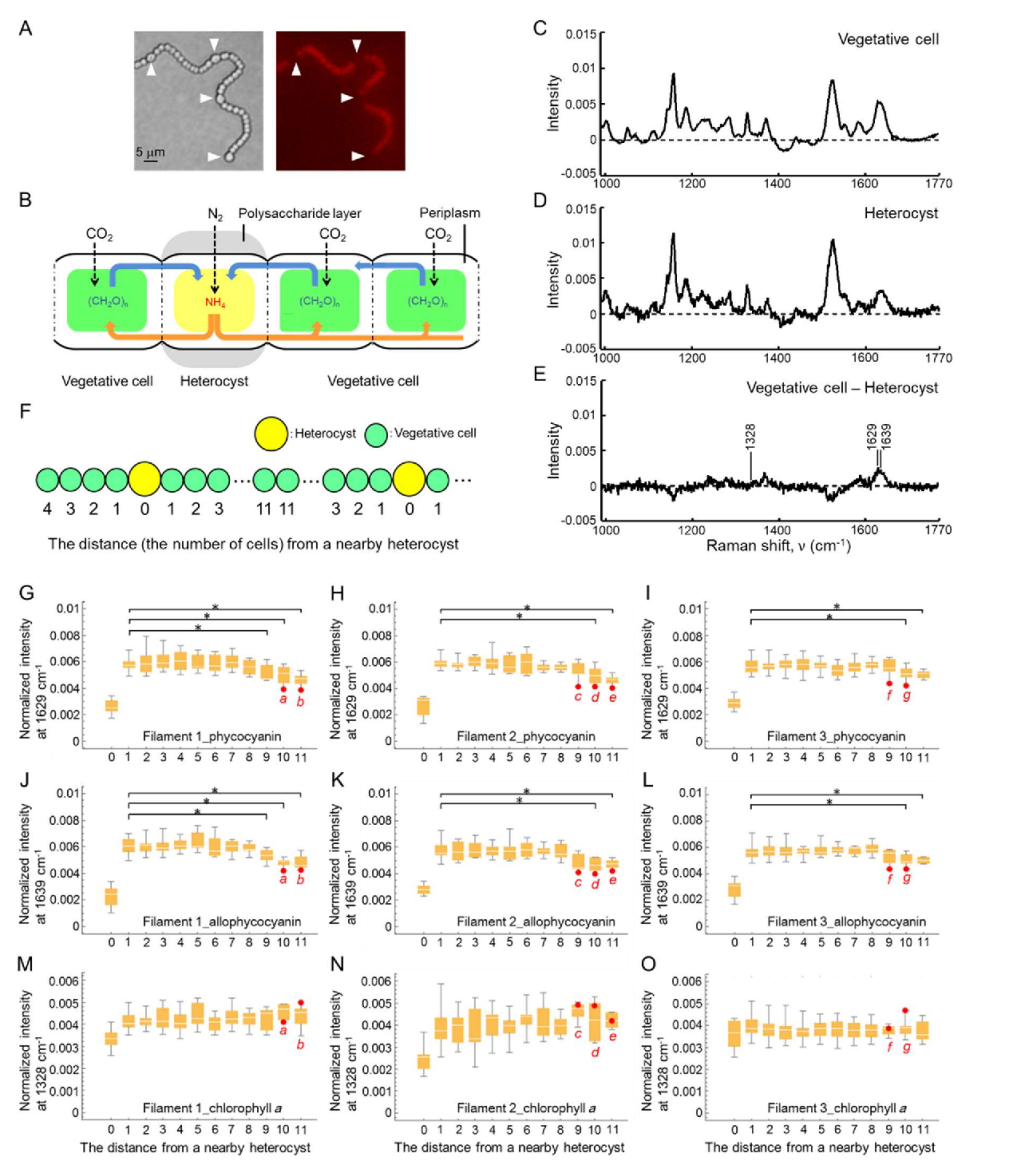
(A) Photomicrographs and schematic representation of *Anabaena* sp. PCC 7120 heterocyst pattern under nitrogen-deprived conditions. Heterocysts are observed as expanded cells with degraded phycobilisome complex. Left and right panels in the photomicrographs show a bright field micrograph and a phycobilisome fluorescence micrograph, respectively. White arrows indicate heterocysts. (B) Heterocysts and neighboring vegetative cells exchange carbohydrates and nitrogen-based compounds through the periplasm surrounding the cells. (C, D) Normalized Raman mean spectra of vegetative cells and heterocysts at an excitation at 785 nm. We measured the Raman spectra of all vegetative cells and heterocysts in a filament (Filament 1) for every single cell. (E) Differences between Raman spectra (C) and (D). The Raman spectrum in Figure D was subtracted from that in Figure C. The Raman bands labeled by arrows (at 1,328, 1,629, and 1,639 cm ^−1^ ) are representative bands assigned to the vibrations of chlorophyll *a* , phycocyanin, and allophycocyanin, respectively. (F) Schematic representation of counting the distance from a nearby heterocyst. In the case of a heterocyst, the distance is counted as 0. Vegetative cells in a filament are numbered along each filament starting from a nearby heterocyst. (G–O) Distributions of the normalized band intensities at 1,629, 1,639, and 1,328 cm ^−1^ at different distances from a nearby heterocyst in the three *Anabaena* filaments (Filaments 1–3) **.** The number of the data points was 108, 120, and 169, which corresponds to the number of cells in Filaments 1–3, respectively. Filaments 1, 2, and 3 included 6, 7, and 10 heterocysts, respectively. Vegetative cells colored in red are the cells *a* – *g* , which showed specifically low intensities at 1,629 and 1,639 cm ^−1^ (intensities were more than twice that of the standard deviation in each filament). In Figures G–I, we performed statistical analyses on the Raman band intensities at 1,629 cm ^−1^ between the cells next to a heterocyst (with a distance of 1) and other cells (with a distance of ≥2) in order. In Figures J–L (and Figures M–O), we also performed statistical analyses on the Raman band intensities at 1,639 (and 1,328) cm ^−1^ in the same manner. Statistical differences are indicated with an * ( *p* < 0.05).

## Description

The filamentous multicellular cyanobacterium, *Anabaena* sp. PCC 7120 (hereafter named *Anabaena* ), is composed of many vegetative cells connected in a one-dimensional manner (Figure A). The vegetative cells are specialized for photosynthesis and contain both Photosystem I (PSI) and Photosystem II (PSII) complexes. Under nitrogen-deprived conditions, some *Anabaena* cells differentiate into cells named heterocysts, which are specialized for nitrogen fixation, at an interval of approximately every ten cells along the filament (Meeks et al., 2002; Golden et al., 2003; Zhang et al., 2006; Kumar et al., 2009; Ishihara et al., 2015) . Once a heterocyst is fully differentiated, it never reverts back to a vegetative cell. As the number of vegetative cells increases via cell division, new heterocysts are differentiated approximately midway between two existing heterocysts. A heterocyst is easily distinguished using a standard light microscope; it is larger in size and rounder in shape than vegetative cells. Additionally, phycobilisome complexes in PSII are chemically decomposed and/or inactivated in heterocysts, leading to suppression of oxygen-generating PSII activity (Zhang et al., 2006) . Heterocysts and vegetative cells exchange metabolites produced by either nitrogen fixation or photosynthesis with neighboring cells, because photosynthesis and nitrogen fixation are incompatible in the same cell (Meeks et al., 2002) (Figure B).

Differences between the photosynthetic systems of vegetative cells and heterocysts have frequently been studied using fluorescence microscopy (Asai et al., 2009; Toyoshima et al., 2009; Sugiura et al., 2012; Ferimazova et al., 2013; Kumazaki et al., 2013) . We previously analyzed microbial pigment compositions in *Anabaena* vegetative cells and heterocysts in a non-invasive and non-labeling manner using Raman spectral measurements (Ishihara et al., 2013; Ishihara et al., 2023) . Fifteen vegetative cells (or heterocysts) were selected randomly from several filaments, and the Raman spectra were measured for every single cell at an excitation at 785 nm. Raman spectral bands were assigned based on the vibrational resonance modes associated with the light-harvesting pigments chlorophyll *a* , phycocyanin, and allophycocyanin. We found the Raman band intensities of phycocyanin and allophycocyanin to be remarkably decreased, particularly in the heterocysts, when compared with those of chlorophyll *a* . Given that phycocyanin and allophycocyanin are components of the light-harvesting phycobilisome complex, our previous study shows good correspondence with earlier studies reporting that phycobilisomes are decomposed during this differentiation (chemical decomposition reduces the amount of the target molecule and its corresponding Raman intensity) (Toyoshima et al., 2009) .

The current study aims to investigate how pigment composition in individual cells is affected by nearby heterocysts. To address this issue, we measured the Raman spectra of individual cells along an *Anabaena* filament by selecting the central points of the cells in a sequential manner. The average Raman spectra of vegetative cells and heterocysts from an entire filament (as an example, Filament 1) are shown in Figures C and D. The procedures we used to obtain the Raman spectra are explained in Methods. The intensity values of Raman spectra in the region of 990 to 1,770 cm ^−1^ were normalized to unity, and the normalized spectra of all vegetative cells (or heterocysts) in the filament were averaged (Figures C and D). The band positions in the Raman spectra of the vegetative cells were nearly the same as those of the heterocysts. Hereafter, normalized band intensities at 1,328, 1,629, and 1,639 cm ^−1^ were selectively used as the signal of chlorophyll *a* , phycocyanin, and allophycocyanin, respectively (Ishihara et al., 2023) . To show specific spectral feature differences more clearly, we subtracted the Raman spectrum in heterocysts from that in vegetative cells (Figure E). The intensities of the Raman bands at 1,629 and 1,639 cm ^−1^ are remarkably higher in the vegetative cells, indicating that these peaks are potential differentiation markers for *Anabaena* .

For the following analysis, we used the Raman spectra of individual cells from three *Anabaena* filaments (Filaments 1–3). The number of vegetative cells enclosed by two heterocysts (“segment length”) was 14.6±6.2, 14.1±6.0, and 14.4±6.6 (avg±s.d.) in Filaments 1–3, respectively. The maximum segment length was 22 in any filaments. Here, the number of vegetative cells away from a nearby heterocyst is referred as “distance” (Figure F). In any filaments, the distance was at most 11 cells. We next plotted the normalized band intensities at 1,629 and 1,639 cm ^−1^ against a particular distance to see quantitative fluctuations of phycocyanin and allophycocyanin in individual cells from the three filaments (Figures G–L). The ranges of the former and latter band intensities among the vegetative cells were 0.0039–0.0079 and 0.0040–0.0076, respectively. Specifically, the upper limit of the band intensity among the heterocysts was lower than the lower limit among the vegetative cells in all filaments tested (Figures G–L). That is, all vegetative cells indicated unique higher band intensities of phycocyanin and allophycocyanin. However, we found that the band intensities at 1,629 and 1,639 cm ^−1^ decrease when vegetative cells are located further from its nearest heterocyst. In the case of Filament 1, these band intensities statistically decreased when the distance was more than 9, as compared with intensities in neighboring heterocyst cells ( *p* < 0.05, Welch’s *t* -test, Figures G and J). In the case of Filaments 2 and 3, the band intensities of phycocyanin and allophycocyanin statistically decreased when the distance was more than 10, as compared with intensities in neighboring heterocyst cells ( *p* < 0.05, Welch’s *t* -test, Figures H, I, K, and L).

Furthermore, we plotted the normalized band intensity at 1,328 cm ^−1^ to see quantitative fluctuations of chlorophyll *a* in the same manner (Figures M–O). The range of band intensities at 1,328 cm ^−1^ was 0.0021–0.0059 among the vegetative cells. As Figures M–O show, the band intensities at 1,328 cm ^−1^ did not statistically decrease when vegetative cells are located far from its nearest heterocyst ( *p* > 0.05, Welch’s *t* -test). The band intensities at 1,328 cm ^−1^ are statistically identical without regard to distance in all filaments ( *p* > 0.05, Welch’s *t* -test, Figures M–O). Thus, we consider that phycocyanin and allophycocyanin decomposition had already begun in vegetative cells located far from its nearest heterocyst.

As Figures G–I show, some vegetative cells (cells *a* – *g* in Figures G–I) showed specifically lower band intensities at 1,629 cm ^−1^ . The intensities at 1,629 cm ^−1^ in cells *a* – *g* were greater than twice those of the standard deviation, which was calculated from the intensities at 1,629 cm ^−1^ in all vegetative cells per each filament. We found that cells *a* – *g* were all located in the intermedium region between two existing heterocysts. Moreover, these same vegetative cells *a* – *g* also showed specifically lower band intensities at 1,639 cm ^−1^ (Figures J–L). Given that the band intensities at 1,328 cm ^−1^ in the vegetative cells *a* – *g* were not specifically lower (Figures M–O), we considered that specific decomposition of phycobilisomes was occurring preferentially in these cells. In fact, we confirmed that cell *c* differentiated into a heterocyst 8 hours later after our Raman spectral measurement. Thus, we consider cell *c* to be a “proheterocyst” cell at the time we measured its Raman spectrum. Proheterocyst cells begin to lose phycobilisome structures, yet are still vegetative cells functionally and morphologically (Asai et al., 2009) . Although we did not observe differentiation in the other cells until 8 hours after the Raman spectral measurement, we were able to observe the specific decomposition of phycobilisomes before the initiation of cellular differentiation using a Raman microscope.

Our current results suggest that the amount of phycocyanin and allophycocyanin do not decrease when a vegetative cell is close to a heterocyst. This is because a nitrogen compound (ammonium ions) is sufficiently supplied to vegetative cells from nearby heterocysts. However, when vegetative cells are deficient of nitrogen compounds, vegetative cells initiate the decomposition of phycobilisomes to gain ammonium ions (Asai et al., 2009; Toyoshima et al., 2009) . In this study, we observed that vegetative cells located far from its nearest heterocyst initiate the decomposition of phycobilisomes before cellular differentiation. In fact, we confirmed that one of the *a* – *g* cells did finally differentiate into a heterocyst. Thus, we were able to observe the decomposition of phycobilisomes in proheterocysts, which precedes those cells becoming candidate heterocysts. In the future, we will be able to potentially predict which cells will differentiate or not by measuring Raman spectra in individual cells.

## Methods

**Bacterial strains and culture**

*Anabaena* sp. PCC 7120 (wild type) were grown in 25 ml of BG-11 _0_ (lacking sodium nitrate) liquid medium at 30℃ under illumination with white fluorescent lamps (FL30SW-B, Hitachi co.) at 45 mM photons m ^-2^ s ^-1^ . The culture was shaken and incubated at 120 rpm until an optimal density at 730 nm (OD _730_ ) reached about 0.4–0.5. The liquid culture was washed three times by using BG11 _0_ liquid medium, diluted to an OD730 of ~0.2, and underlain beneath a fresh BG-11 _0_ solid medium plate containing 1.5 % agar solution (Becton, Dickinson and company, USA) with a bottom dish glass. The sample was placed in a Raman microscope (as mentioned below) kept at 30℃ under illumination with white fluorescent lamps at 45 mM photons m ^-2^ s ^-1^ .

**Raman microscope and spectral pre-treatments**

In Via confocal Raman spectrometer equipped with a CCD camera (inVia Reflex, Renishaw co.) was used to measure the Raman spectrum. The excitation wavelength was at 785 nm. We measured Raman spectra of individual vegetative cells and heterocysts by selecting the central points of the cells. A typical Raman spectrum of a small confocal volume in the cytoplasm of a single living vegetative cell yields a sufficient signal-to-noise ratio for analysis (~1 s per pixel, with a 785 nm laser at ~20 mW directed at the confocal volume). In this study, we corrected the baselines of Raman spectra. The baseline-corrected Raman spectrum *y* ’( *ν* ) was calculated as *y* ’( *ν* ) = *y* ( *ν* )- *y*_poly_ ( *ν* ), in which *y*_poly_ ( *ν* ) is a fitted polynomial curve constructed by the following procedures. (i) For a spectrum truncated between the minimum Raman shift position *ν*_min_ and the maximum position *ν*_max_ , we selected the degree of the function *d* to fit the baseline using a polynomial function ( *d* =3). (ii) Using the least squares method, we first fitted the polynomial function *y*_poly_ to the Raman spectrum *y* . (iii) We divided the Raman spectrum *y* into upper and lower parts, relative to the fitted baseline *y*_poly_ . (iv) The number of data points on the upper side of *y* was defined as *N*_A_ , and the number on the lower side of *y* was defined as *N*_B_ . If *N*_A_ < *N*_B_ , we removed the upper part of *y* from the whole of *y* , and we replaced the Raman spectrum *y* with the lower part of the spectrum. Then, we repeated the procedure (ii). When *N*_A_ ≥ *N*_B_ , the baseline was considered the best fit and optimal.

**Statistical analysis**

Welch’s *t* -test was used to determine differences between the Raman band intensities at 1,629 cm ^−1^ between the cells next to a heterocyst (with a distance of 1) and other cells (with a distance of ≥2) in order. We also performed statistical analyses on the Raman band intensities at 1,639 (and 1,328) cm ^−1^ in the same manner. Statistical difference was considered if *p* < 0.05. All statistical analysis was carried out in Mathematica version 10.
